# Maternal and perinatal complications according to maternal age: A systematic review and meta‐analysis

**DOI:** 10.1002/ijgo.14100

**Published:** 2022-02-07

**Authors:** Gabriele Saccone, Elisabetta Gragnano, Bernadette Ilardi, Vincenzo Marrone, Ida Strina, Roberta Venturella, Vincenzo Berghella, Fulvio Zullo

**Affiliations:** ^1^ Department of Neuroscience, Reproductive Sciences and Dentistry, School of Medicine University of Naples Federico II Naples Italy; ^2^ Division of Obstetrics and Gynecology University of Catanzaro Catanzaro Italy; ^3^ Division of Maternal‐Fetal Medicine, Department of Obstetrics and Gynecology Sidney Kimmel Medical College of Thomas Jefferson University Philadelphia Pennsylvania USA

**Keywords:** advanced maternal age, ART, maternal mortality, pregnancy

## Abstract

**Objective:**

To evaluate the risk levels for maternal and perinatal complications at > 40, > 45 and > 50 years old compared with younger controls.

**Methods:**

Electronic databases were searched from their inception until March 2021. We included studies reporting pregnancy outcome in pregnant women aged 40, 45, and 50 years or older compared with controls at the time of delivery. Case reports and case series were excluded. The primary outcome was the incidence of stillbirth. Meta‐analysis was performed using the random effects model of DerSimonian and Laird, to produce summary treatment effects in terms of relative risk (RR) with 95% confidence interval (CI). Heterogeneity was measured using *I* ^2^ (Higgins *I* ^2^). Subgroup analyses in women older than 45 years and in those older than 50 years were performed.

**Results:**

Twenty‐seven studies, including 31 090 631 women, were included in the meta‐analysis. The overall quality of the included studies was moderate to high. Most of the included studies were retrospective cohort studies (21/27), four were population‐based studies, and two were cross‐sectional studies. Women aged ≥40 years had significantly higher risk of stillbirth (RR 2.16, 95% CI 1.86–2.51), perinatal mortality, intrauterine growth restriction, neonatal death, admission to neonatal intensive care unit, pre‐eclampsia, preterm delivery, cesarean delivery, and maternal mortality compared with women younger than 40 years old (RR 3.18, 95% CI 1.68–5.98). The increased risks for maternal mortality were 42.76 and 11.60 for women older than 50 years and for those older than 45 years, respectively, whereas those for stillbirth were 3.72 and 2.32. The risk of stillbirth and cesarean delivery was significantly higher in women >45 years compared with those aged 40–45 years, and in those aged >50 years compared with those aged 45–50 years. The risk of maternal mortality was significantly higher in women aged >50 years compared with those aged 40–45 (RR 60.40, 95% CI 13.28–274.74).

**Conclusion:**

The risk of stillbirth, cesarean delivery, and maternal mortality increases with advancing maternal age. The risk ratios for maternal mortality were 3.18, 11.60, and 42.76 in women older than 40, older than 45, and older than 50 years, respectively. These data should be used when women with advanced maternal age are counseled regarding their risk in pregnancy.

**Systematic Review Registration:**

The review was registered with the PROSPERO International Prospective Register of Systematic Reviews (registration No.: CRD42020208788).

## 1 INTRODUCTION

The trend of deferring childbirth to a later time in a woman’s life is associated with an increased risk of infertility and the use of assisted reproductive technologies, including in vitro fertilization, intracytoplasmic sperm injection, or oocyte donation. Oocyte donation enables women with diseases such as premature ovarian insufficiency, genetic disorders, or surgical menopause to become pregnant.^1^ The technique is also used to overcome natural perimenopausal or postmenopausal infertility, making motherhood possible for women even in their sixties.^2^ Several studies have shown that assisted reproductive technologies (ART), including in vitro fertilization, intracytoplasmic sperm injection, or oocyte donation, are associated with an increased risk of maternal and perinatal complications compared with spontaneously conceived pregnancies.^3^, ^4^, ^5^, ^6^, ^7^, ^8^, ^9^, ^10^, ^11^

Advanced maternal age, traditionally referred to pregnant women aged 35 years or older at the time of delivery, is associated with an increased risk of maternal and perinatal complications among singleton and multiple gestations.^12^, ^13^ The risk seems even higher in women aged 40 years or older,^14^ but the literature is inconsistent and limited to retrospective data.

To address this inconsistency in knowledge, the aim of this systematic review was to evaluate the risk levels for maternal and perinatal complications at ≥40, ≥45, and > 50 years of age compared with younger controls.

## 2 MATERIALS AND METHODS

### 2.1 Search strategy and selection criteria

This review was performed according to a protocol designed a priori and recommended for systematic review.^14^ Electronic databases (i.e., MEDLINE, Scopus, ClinicalTrials.gov, EMBASE, Sciencedirect, the Cochrane Library at the CENTRAL Register of Controlled Trials, Scielo) were searched from their inception until March 2021. Search terms used were the following text words: “maternal age”, “advanced”, “pregnancy”, and “outcome” combined. No restriction for geographic location was applied. Only studies published in the English language were included. The search was restricted to publication year 2000 and later. In addition, the reference lists of all identified articles were examined to identify studies not captured by electronic searches. The electronic search and the eligibility of the studies were independently assessed by two authors (GC, GS). Differences were discussed and consensus was reached.

We included all cohort studies reporting pregnancy outcome in pregnant women older than 40, 45, and 50 years compared with controls. Case–control studies, case reports, and case series were excluded. Studies published only as abstract were also excluded.

### 2.2 Primary and secondary outcomes

Primary and secondary outcomes were defined before data extraction. The primary outcome was the incidence of stillbirth. The secondary outcomes were perinatal mortality, neonatal death, admission to neonatal intensive care unit (NICU), preterm birth, cesarean delivery, and maternal mortality. When possible, data on use of ART were extracted. Subgroup analyses according to women older than 45 years and older than 50 years were performed. We also planned to perform indirect meta‐analyses to compare risk of primary outcome (i.e., stillbirth), cesarean delivery, and maternal mortality according to maternal age at different cut‐offs (40–45, >45, and >50 years).

### 2.3 Study definition

Stillbirth was defined as intrauterine fetal death according to individual study gestational age cut‐off. Pre‐eclampsia was defined as blood pressure >140/90 mm Hg with significant proteinuria or as classified by authors where definition was not provided. Intrauterine growth restriction was defined as estimated fetal weight below the 10th centile adjusted for gestational age or related definitions specified by the original study. Neonatal death was defined as the death of a liveborn infant, regardless of gestational age at birth, within the first 28 completed days of life. Perinatal mortality was defined as either stillbirth or neonatal death. Preterm birth was defined as delivery before 37 weeks of gestation.^15^

### 2.4 Statistical analysis

Data extraction and data analysis were completed independently by two authors (VDV, GS) using review manager v. 5.3 (The Nordic Cochrane Centre, Cochrane Collaboration, 2014). The completed analyses were then compared, and any difference was resolved by discussion.

Data from each eligible study were extracted without modification of original data onto custom‐made data collection forms. For dichotomous variables, a 2‐by‐2 table was assessed, and relative risk (RR) was computed. For continuous outcomes, means ± standard deviation were extracted and imported into review manager v. 5.3, and mean difference (MD) was calculated.

Meta‐analysis was performed using the random effects model of DerSimonian and Laird, to produce summary treatment effects in terms of either an RR or MD with 95% confidence interval (CI). Heterogeneity was measured using *I* ^2^ (Higgins *I* ^2^). A *P* value less than 0.05 was considered statistically significant.

The meta‐analysis was reported following the Preferred Reporting Item for Systematic Reviews and Meta‐analyses (PRISMA) statement.^16^

Before data extraction, the review was registered with the PROSPERO International Prospective Register of Systematic Reviews (registration no. CRD42020208788).

## 3 RESULTS

### 3.1 Study selection and study characteristics

**FIGURE 1:**
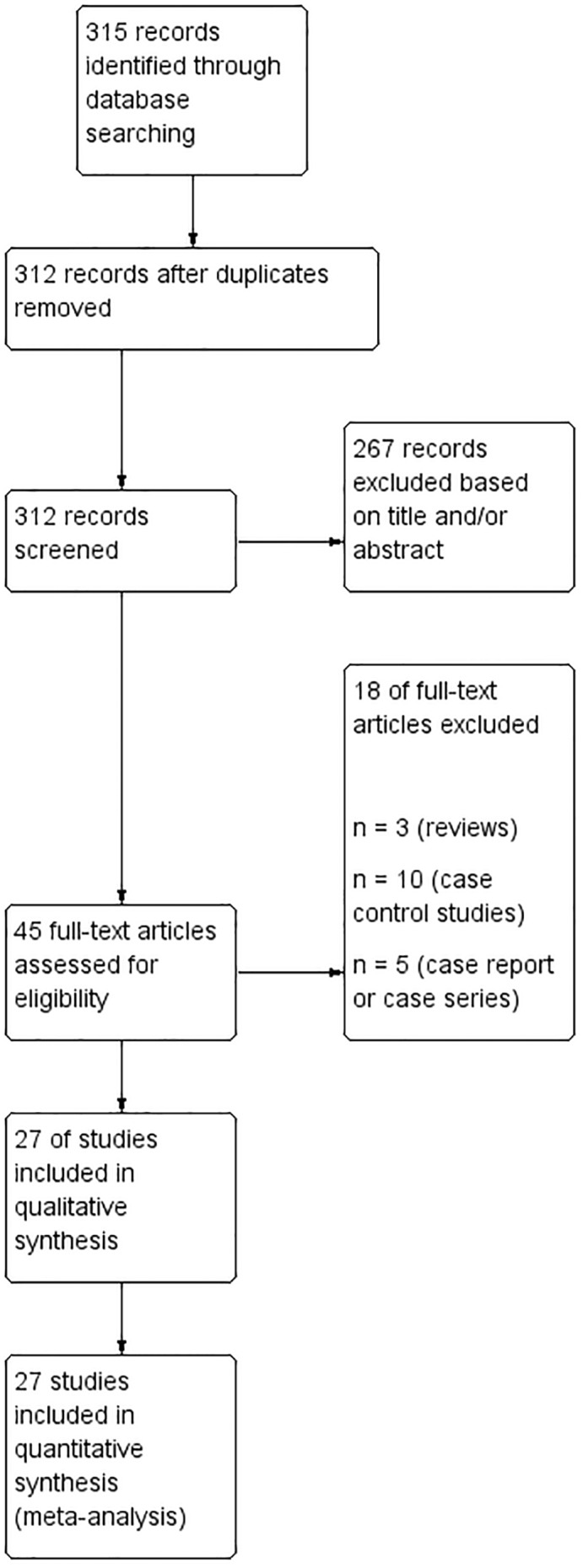
Flow diagram of studies identified in the systematic review. (PRISMA template [preferred reporting item for systematic reviews and meta‐analyses])

**FIGURE 2:**
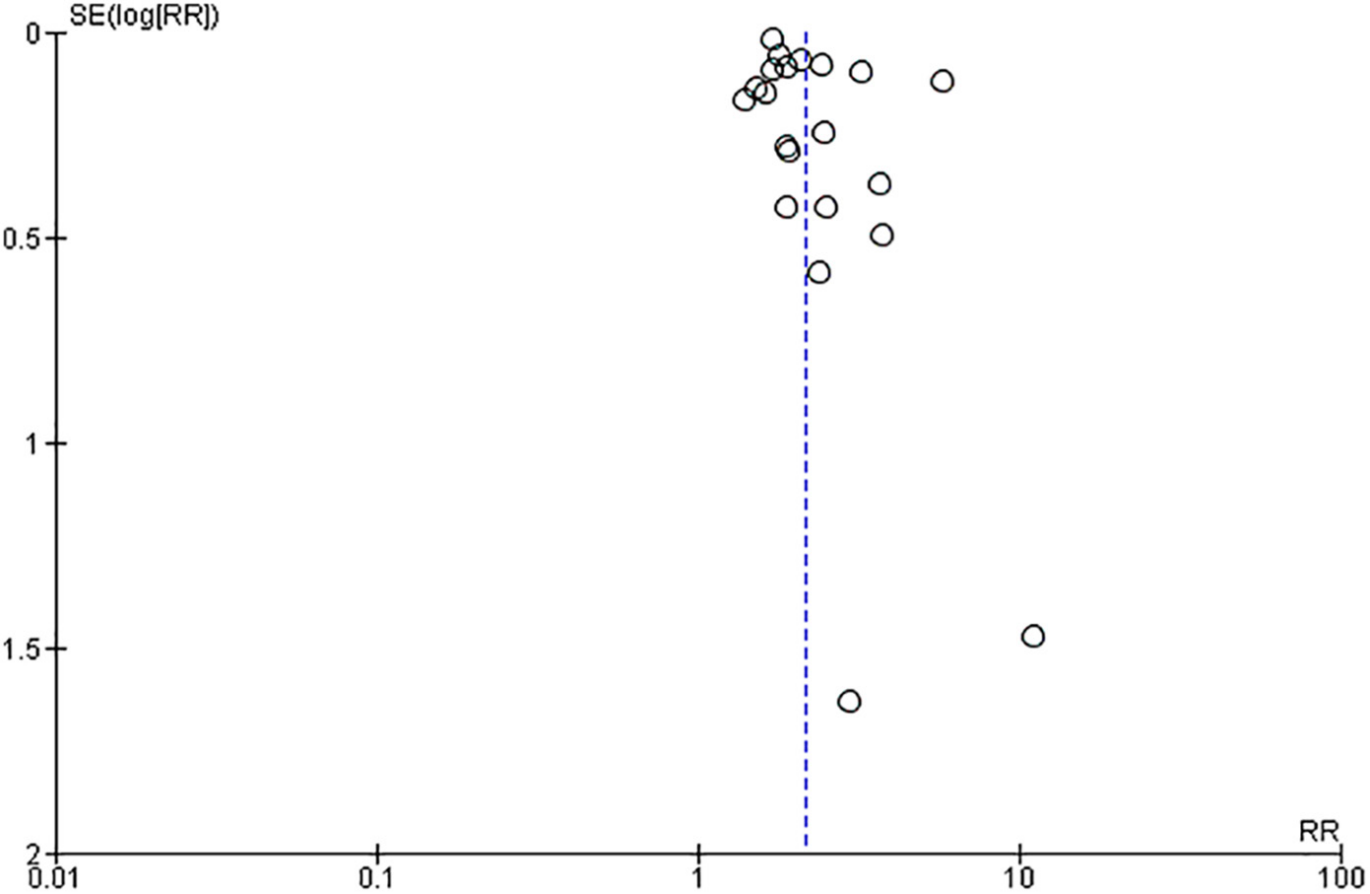
Funnel plot for publication bias

Twenty‐seven studies were included in the meta‐analysis^17^, ^18^, ^19^, ^20^, ^21^, ^22^, ^23^, ^24^, ^25^, ^26^, ^27^, ^28^, ^29^, ^30^, ^31^, ^32^, ^33^, ^34^, ^35^, ^36^, ^37^, ^38^, ^39^, ^40^, ^41^, ^42^, ^43^ (Figure 1). Overall, 31 090 631 participants were included in the review. Of them 733 327 were women older than 40 years, and 30 357 304 were women younger than 40 years (Table 1). The overall quality of the included studies was moderate to high. The vast majority of the included studies were retrospective cohort studies (21/27), four were population‐based studies, and two were cross‐sectional studies. Publication bias, assessed by visual inspection of funnel plot (Figure 2), showed no publication bias.

**TABLE 1:** Characteristics of the included studies

	Study location	Study group, year	Control group, year	Assisted reproductive technology	Study design
Abu 2000^17^	Jordan	>45 (*n* = 114)	20–29 (*n* = 121)	Not reported	Retrospective cohort
Canterino 2004^18^	USA	40–44 (*n* = 424 820)45–49 (*n* = 16 739)	15–19 (*n* = 2 728 602) 20–24 (*n* = 5 440 685) 25–29 (*n* = 6 000 811) 30–34 (*n* = 4 970 770) 35–39 (*n* = 2 028 446)	Not reported	Retrospective cohort
Jacobsson 2004^19^	Sweden	40–44 (*n* = 31 662) > 45 (*n* = 1205)	20–29 (*n* = 876 361)	Not reported	Population‐based
Dieijomaoh 2006^20^	Kuwait	40–47 (*n* = 168)	25–30 (*n* = 160)	Not reported	Retrospective cohort
Hoffman 2007^21^	Miami, FL, USA	>40 (*n* = 3953)	<35 (*n* = 108 547) 35–39 (*n* = 13 902)	Not reported	Retrospective cohort
Donoso 2008^22^	Chile	>50 (*n* = 217)	20–34 (*n* = 2 817 742)	Not reported	Population‐based
Jahromi 2008^23^	Iran	>40 (*n* = 200)	20–30 (*n* = 200)	Not reported	Retrospective cohort
Salihu 2008^24^	Missouri, USA	>40 (*n* = 13 453)	20–24 (*n* = 429 647) 25–29 (*n* = 441 718) 30–34 (*n* = 265 167) 35–39 (*n* = 85 322)	Not reported	Retrospective cohort
Hsieh 2010^25^	Taiwan	>40 (*n* = 721)	20–34 (*n* = 33 881) 35–39 (*n* = 5161)	20–34 (*n* = 624) 35–39 (*n* = 243) > 40 (*n* = 24)	Retrospective cohort
Arnold 2012^26^	Australia	>40 (*n* = 2148)	<40 (*n* = 60 203)	<40 (*n* = 2820) > 40 (*n* = 323)	Retrospective cohort
Ates 2012^27^	Turkey	>40 (*n* = 97)	20–29 (*n* = 97)	Not reported	Population‐based
Favilli 2012^28^	Italy	>40 (*n* = 317)	20–30 (*n* = 312)	20–30 (*n* = 0) > 40 (*n* = 10)	Retrospective cohort
Kenny 2013^29^	UK	>40 (*n* = 7066)	20–29 (*n* = 122 307) 30–34 (*n* = 62 371) 35–39 (*n* = 33 966)	Not reported	Population‐based
Khalil 2013^30^	UK	>40 (*n* = 4061)	<35 (*n* = 55 772) 35–39 (*n* = 16 325)	Not reported	Retrospective cohort
Ngowa 2013^31^	Cameroon	>40 (*n* = 585)	20–29 (*n* = 1816)	Not reported	Retrospective cohort
Seckin 2013^32^	Turkey	>40 (*n* = 190)	20–30 (*n* = 600)	Not reported	Retrospective cohort
Timofeev 2013^33^	Washington, USA	40–45 (*n* = 5931) > 45 (*n* = 391)	<20 (*n* = 19 638) 20–24 (*n* = 51 011) 25–30 (*n* = 56 480) 31–34 (*n* = 45 715) 35–39 (*n* = 24 351)	Not reported	Retrospective cohort
Laopiboon 2014^34^	Thailand	40–44 (*n* = 7015) > 45 (*n* = 1527)	20–34 (*n* = 238 504) 35–39 (*n* = 29 245)	Not reported	Cross‐sectional
Mutz 2014^35^	Austria	>40 (*n* = 2272)	25–34 (*n* = 43 313) 35–39 (*n* = 10 932)	Not reported	Retrospective cohort
Waldenstrom 2014^36^	Sweden, and Norway	>40 (*n* = 11 430)	25–29 (*n* = 342 012) 30–34 (*n* = 222 883) 35–39 (*n* = 67 859)	Not reported	Retrospective cohort
Traisrislip 2015^37^	Thailand	>40 (*n* = 797)	20–30 (*n* = 18 802)	Not reported	Retrospective cohort
Goisis 2017^38^	Finland	>40 (*n* = 2903)	10–19 (*n* = 2183) 20–24 (*n* = 20 562) 25–29 (*n* = 45 946) 30–34 (*n* = 37 580) 35–39 (*n* = 14 924)	Not reported	Retrospective cohort
Marozio 2017^39^	Italy	40–44 (*n* = 3541) > 45 (*n* = 257)	<40 (*n* = 52 413)	<40 (*n* = 1704) 40–44 (*n* = 280) > 45 (*n* = 61)	Retrospective cohort
Ogawa 2017^40^	Japan	40–44 (*n* = 28 797) > 45 (*n* = 924)	30–34 (*n* = 204 181) 35–39 (*n* = 131 515)	30–34 (*n* = 4963) 35–39 (*n* = 8641) 40–44 (*n* = 3987) > 45 (*n* = 201)	Cross sectional study
Frederiksen 2018^41^	Denmark	>40 (*n* = 9743)	20–34 (*n* = 300 863) 35–39 (*n* = 58 910)	20–34 (*n* = 15 515) 35–39 (*n* = 6877) > 40 (*n* = 1898)	Retrospective cohort
Rydahl 2019^42^	Denmark	>40 (*n* = 31 361)	<30 (*n* = 517 450) 30–34 (*n* = 398 873) 35–39 (*n* = 175 280)	Not reported	Retrospective cohort
Rademaker 2020^43^	Netherlands	40–44 (*n* = 112 952) 45–49 (*n* = 4631) > 50 (*n* = 157)	25–29 (*n* = 1 085 447)	25–29 (*n* = 5916) 40–44 (*n* = 4543) 45–49 (*n* = 290) > 50 (*n* = 51)	Retrospective cohort

Regarding the study group, it was >40 in 23 studies,^19^, ^21^, ^23^, ^24^, ^25^, ^26^, ^27^, ^28^, ^29^, ^30^, ^31^, ^32^, ^33^, ^34^, ^35^, ^36^, ^37^, ^38^, ^39^, ^40^, ^41^, ^42^, ^43^ >45 in one study,^17^ 40–49 in one study,^18^ >50 in one study,^22^ and 40–47 in one study.^20^ Most of studies reported subgroup analyses according to different age cut‐offs in both study group and control group.

### 3.2 Synthesis of results

**FIGURE 3:**
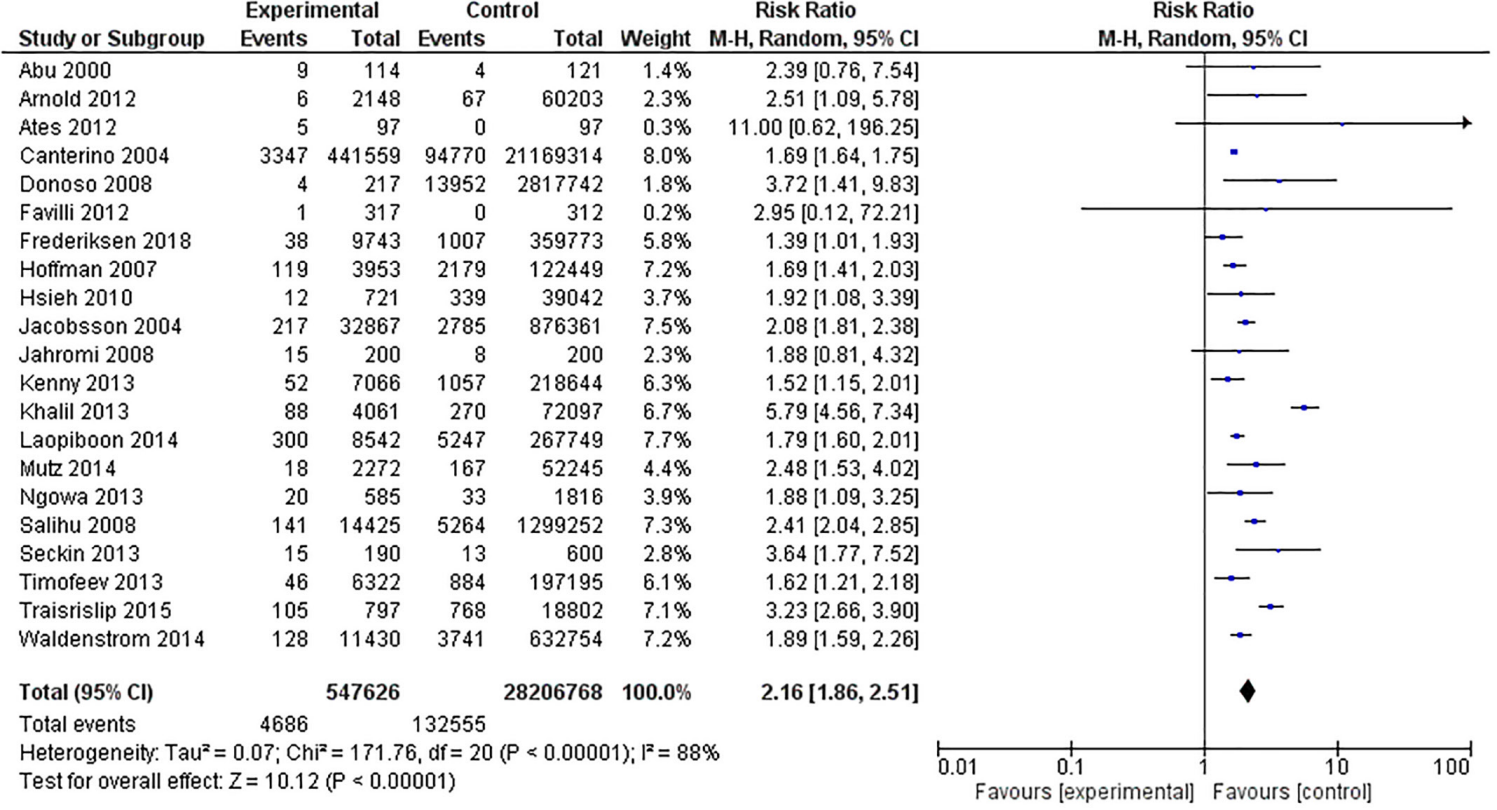
Forest plot for the risk of stillbirth in women older than 40 years

**FIGURE 4:**
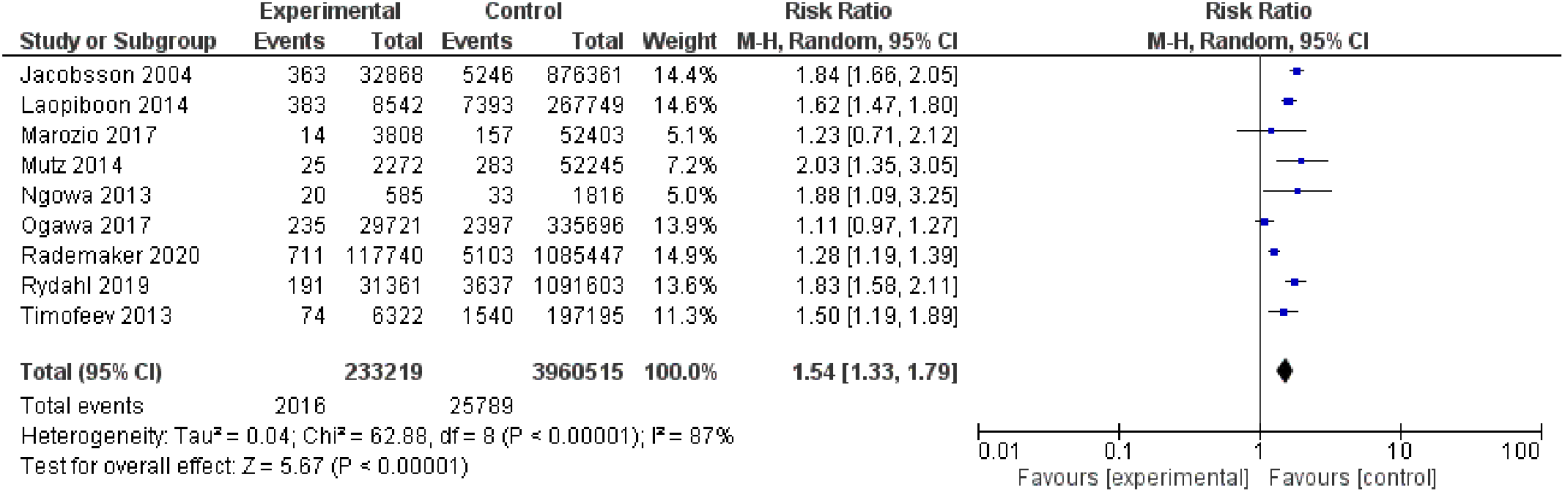
Forest plot for the risk of perinatal death in women older than 40 years

**FIGURE 5:**
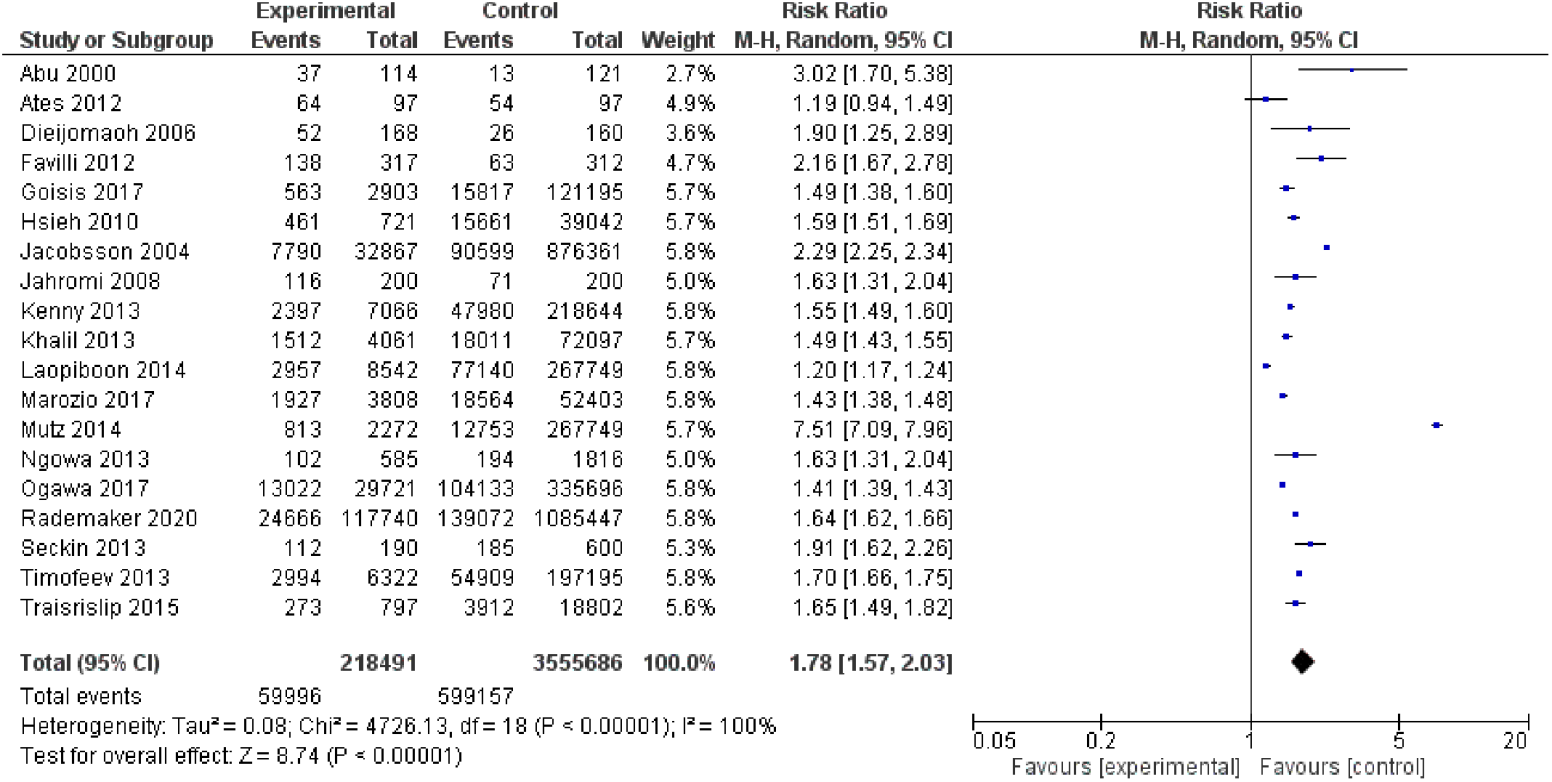
Forest plot for the risk of cesarean delivery in women older than 40 years

Table 2 shows the primary and secondary outcomes in the overall analysis. Women older than 40 years had significantly higher risk of stillbirth (Figure 3), perinatal mortality (Figure 4), intrauterine growth restriction, neonatal death, admission to NICU, pre‐eclampsia, preterm delivery, cesarean delivery (Figure 5), and maternal mortality. Increased risks of maternal and perinatal complications were still significant in the group of women older than 45 years (Table 3).

**TABLE 2:** Primary and secondary outcome in the overall analysis^a^ ^,^ ^b^

	Sample size	Stillbirth	Perinatal mortality	IUGR	Neonatal death	NICU admission	Pre‐eclampsia	Preterm birth	Cesarean delivery	Maternal mortality
Abu 2000^17^	114 vs 121	9/114 vs 4/121	NR	3/114 vs 1/121	4/114 vs 1/121	NR	14/114 vs 2/121	9/114 vs 6/121	37/114 vs 13/121	NR
Canterino 2004^18^	441 559 vs 21 169 314	3347/441 559 vs 94 770/21 169 314	NR	NR	NR	NR	NR	NR	NR	NR
Jacobsson 2004^19^	32 867 vs 876 361	217/32 867 vs 2785/876 361	363/32 867 vs 5246/876 361	NR	146/32 867 vs 2461/876 361	NR	775/32 867 vs 25 547/876 361	2875/32 867 vs 54 309/876 361	7790/32 867 vs 90 599/876 361	NR
Dieijomaoh 2006^20^	168 vs 160	NR	NR	4/168 vs 1/160	NR	NR	NR	NR	52/168 vs 26/160	NR
Hoffman 2007^21^	3953 vs 122 449	119/3953 vs 2179/122 449	NR	NR	NR	NR	368/3953 vs 7491/122 449	NR	NR	NR
Donoso 2008^22^	217 vs 2 281 774	4/217 vs 13 952/2 281 774	NR	NR	13/217 vs 17 396/2 281 774	NR	NR	NR	NR	0/217 vs 686/2 281 774
Jahromi 2008^23^	200 vs 200	15/200 vs 8/200	NR	NR	NR	NR	36/200 vs 9/200	62/200 vs 38/200	116/200 vs 71/200	NR
Salihu 2008^24^	14,425 vs 1 299 252	141/14 425 vs 5264/1 299 252	NR	NR	NR	NR	NR	NR	NR	NR
Hsieh 2010^25^	721 vs 39 042	12/721 vs 339/39 042	NR	NR	8/721 vs 307/39 042	71/721 vs 2,482/39,042	NR	151/721 vs 4,738/39,042	461/721 vs 15,661/39,042	NR
Arnold 2012^26^	2148 vs 60 203	6/2148 vs 67/60 203	NR	NR	NR	NR	NR	NR	NR	NR
Ates 2012^27^	97 vs 97	5/97 vs 0/97	NR	NR	NR	5/97 vs 1/97	NR	15/97 vs 10/97	64/97 vs 54/97	NR
Favilli 2012^28^	317 vs 312	1/317 vs 0/312	NR	NR	NR	1/317 vs 1/312	NR	NR	138/317 vs 63/312	NR
Kenny 2013^29^	7066 vs 218 644	52/7066 vs 1057/218 644	NR	NR	16/7066 vs 485/218 644	NR	NR	564/7066 vs 15 600/218 644	2397/7066 vs 47 980/218 644	NR
Khalil 2013^30^	4061 vs 72 097	88/4061 vs 270/72 097	NR	NR	NR	NR	130/4061 vs 1568/72 097	91/4061 vs 1190/72 097	1512/4061 vs 18 011/72 097	NR
Ngowa 2013^31^	585 vs 1816	20/585 vs 33/1816	20/585 vs 33/1,816	NR	NR	82/585 vs 218/1816	13/585 vs 14/1816	69/585 vs 167/1816	102/585 vs 194/1816	NR
Seckin 2013^32^	190 vs 600	15/190 vs 13/600	NR	19/190 vs 30/600	12/190 vs 16/600	18/190 vs 46/600	30/190 vs 22/600	55/190 vs 120/600	112/190 vs 185/600	NR
Timofeev 2013^33^	6322 vs 197 195	46/6322 vs 884/197 195	74/6,322 vs 1,540/197,195	NR	28/6322 vs 656/197 195	966/6322 vs 24 261/197 195	NR	NR	2994/6322 vs 54 909/197 195	NR
Laopiboon 2014^34^	8542 vs 267 749	300/8542 vs 5247/267 749	383/8,542 vs 7,393/267,749	NR	NR	705/8542 vs 16 542/267 749	NR	650/8542 vs 16 316/267 749	2957/8542 vs 77 140/267 749	NR
Mutz 2014^35^	2272 vs 52 245	18/2272 vs 167/52 245	25/2,272 vs 283/52,245	NR	7/2272 vs 116/52 245	NR	NR	287/2272 vs 4247/52 245	813/2272 vs 12 753/52 245	NR
Waldenstrom 2014^36^	11 430 vs 632 754	128/11 430 vs 3741/632 754	NR	NR	52/11 430 vs 1951/632 754	NR	NR	1338/11 430 vs 59 315/632 754	NR	NR
Traisrislip 2015^37^	797 vs 18 802	105/797 vs 768/18 802	NR	68/797 vs 1045/18 802	NR	NR	NR	280/797 vs 4323/18 802	273/797 vs 3912/18 802	NR
Goisis 2017^38^	2903 vs 121 195	NR	NR	NR	NR	NR	NR	171/2903 vs 4325/121 195	563/2903 vs 15 817/121 195	NR
Marozio 2017^39^	3808 vs 52 403	NR	14/3808 vs 157/52 403	NR	NR	NR	163/3808 vs 1241/52 403	368/3808 vs 2779/52 403	1927/3808 vs 18 564/52 403	NR
Ogawa 2017^40^	29 721 vs 335 696	NR	235/29 721 vs 2397/335 696	NR	NR	NR	1601/29 721 vs 12 774/335 696	5602/29 721 vs 57 319/335 696	13 022/29 721 vs 104 133/335 696	NR
Frederiksen 2018^41^	9743 vs 359 773	38/9743 vs 1007/359 773	NR	NR	NR	NR	NR	178/9743 vs 4274/359 773	NR	NR
Rydahl 2019^42^	31 361 vs 1 091 603	NR	191/31 361 vs 3637/1 091 603	NR	NR	NR	1160/31 361 vs 30 859/1 091 603	NR	NR	NR
Rademaker 2020^43^	117 740 vs 1 085 447	NR	711/117 740 5103/1 085 447	NR	NR	4528/117 740 vs 28 905/1 085 447	NR	9156/117 740 vs 76 043/1 085 447	24 666/117 740 vs 139 072/1 085 447	12/117 740 vs 37/1 085 447
Total	733 327 vs 30 357 304	4686/547 626 (0.85%) vs 132 555/28 206 768 (0.46%)	2016/233 219 (0.86%) vs 25 789/3 960 515 (0.65%)	94/1269 (7.41%) vs 1077/19 683 (5.47)	286/61 199 (0.47%) vs 23 389/ 4 834 704 (0.48%)	6379/134 514 (4.74%) vs 72 456/ 15 922 258 (4.55%)	4290/106 860 (4.01%) vs 79 527/2 553 346 (3.11%)	21 921/232 857 (9.41%) vs 305 119/4 135 042 (7.38%)	59 996/218 491 (27.46%) vs 599 157/3 555 686 (16.85%)	12/117 957 (0.01%) vs 723/3 903 189 (0.01%)
RR (95% CI)	**–**	**2.16 (1.86–2.51)**	**1.54 (1.33–1.79)**	**1.62 (1.31–2.01)**	**1.88 (1.28–2.75)**	**1.34 (1.24–1.46)**	**1.66 (1.35–2.04)**	**1.39 (1.29–1.50)**	**1.78 (1.57–2.03)**	**3.18 (1.68–5.98)**
*I* ^2^	**–**	88%	100%	0%	85%	73%	96%	95%	100%	0%

Abbreviations: CI, confidence interval; IUGR, intrauterine growth restriction; NICU, neonatal intensive care unit; NR, not reported; RR, relative risk.

^a^ Data are presented as number in the group of women >40 years versus number in the group of women <40 years.

^b^ Boldface data are statistically significant.

**TABLE 3:** Primary and secondary outcome in subgroup analysis in women >45 years^a,b^

	Stillbirth	Perinatal mortality	IUGR	Neonatal death	NICU admission	Preeclampsia	Preterm birth	Cesarean delivery	Maternal mortality
Abu 2000^17^	9/114 vs 4/121	NR	3/114 vs 1/121	4/114 vs 1/121	NR	14/114 vs 2/121	9/114 vs 6/121	37/114 vs 13/121	NR
Canterino 2004^18^	120/16,739 vs 94 770/21 169 314	NR	NR	NR	NR	NR	NR	NR	NR
Jacobsson 2004^19^	14/1205 vs 2785/876 361	17/1205 vs 5246/876 361	NR	6/1205 vs 2461/876 361	NR	26/1205 vs 25 547/876 361	113/1205 vs 54 309/876 361	365/1205 vs 90 599/876 361	NR
Donoso 2008^22^	4/217 vs 13 952/2 281 774	NR	NR	13/217 vs 17 396/2 281 774	NR	NR	NR	NR	0/217 vs 686/2 281 774
Timofeev 2013^33^	5/391 vs 884/197 195	7/391 vs 1540/197 195	NR	2/391 vs 656/197 195	70/391 vs 24 261/197 195	NR	NR	220/391 vs 54 909/197 195	NR
Laopiboon 2014^34^	65/1527 vs 5247/267 749	80/1527 vs 7393/267 749	NR	NR	72/1527 vs 16 542/267 749	NR	102/1527 vs 16 316/267 749	348/1527 vs 77 140/267 749	NR
Marozio 2017^39^	NR	1/257 vs 157/52 403	NR	NR	NR	17/257 vs 1241/52 403	48/257 vs 2779/52 403	138/257 vs 18 564/52 403	NR
Ogawa 2017^40^	NR	11/924 vs 2397/335 696	NR	NR	NR	73/924 vs 12 774/335 696	192/924 vs 57 319/335 696	506/924 vs 104 133/335 696	NR
Rademaker 2020^43^	NR	35/4788 vs 5103/1 085 447	NR	NR	284/4788 vs 28 905/1 085 447	NR	504/4788 vs 76 043/1 085 447	1276/4788 vs 139 072/1085 447	2/4788 vs 37/1 085 447
Total	217/20 193 vs 117 642/25 328 482	151/9092 vs 21 836/2 814 851	3/114 vs 1/121	25/1927 vs 20 514/3 891 419	426/6706 vs 69 708/1 550 391	130/2500 vs 39 564/1 264 581	968/8815 vs 206 772/2 617 777	2890/9206 vs 484 430/2 814 972	2/5005 vs 723/3 903 189
RR (95% CI)	**2.32 (1.71–3.16)**	**1.85 (1.58–2.17)**	3.18 (0.34–30.17)	**3.54 (2.31–5.44)**	1.36 (0.72–2.58)	**1.59 (1.34–1.90)**	**1.58 (1.23–2.03)**	**1.82 (1.36–2.42)**	**11.60 (3.27–41.11)**
*I* ^2^	64%	0%	Not applicable	84%	97%	90%	92%	99%	0%

Abbreviations: CI, confidence interval; IUGR, intrauterine growth restriction; NICU, neonatal intensive care unit; NR, not reported; RR, relative risk.

^a^ Data are presented as number in the group of women >45 years versus number in the group of women <40 years.

^b^ Boldface data are statistically significant.

Table 4 shows the primary and secondary outcomes in women older than 50 years. Pooled data from the two included studies^22^, ^43^ showed that women older than 50 years had significantly higher risk of stillbirth (RR 3.72, 95% CI 1.42–9.83), perinatal mortality, neonatal death, admission to NICU, preterm birth, cesarean delivery, and maternal mortality (RR 42.76, 95% CI 12.36–147.92) compared with women younger than 40 years.

**TABLE 4:** Primary and secondary outcome in subgroup analysis in women >50 years^a^ ^,^ ^b^

	Stillbirth	Perinatal mortality	Neonatal death	NICU admission	Preterm birth	Cesarean delivery	Maternal mortality
Donoso 2008^22^	4/217 vs 13 952/2 281 774	NR	13/217 vs 17 396/2 281 774	NR	NR	NR	0/217 vs 686/2 281 774
Rademaker 2020^43^	NR	4/157 vs 5103/1 085 447	NR	19/157 vs 28 905/1 085 447	41/157 vs 76 043/1 085 447	73/157 vs 139 072/1 085 447	2/157 vs 37/1 085 447
Total	4/217 (1.84%) vs 13,952/2 281 774 (0.50%)	4/157 (2.54%) vs 5103/1 085 447 (0.47%)	13/217 (5.60%) vs 17 396/2 2 81 774 (0.62%)	19/157 (12.10%) vs 28 905/1 085 447 (2.66%)	41/157 (26.11%) vs 76 043/1 085 447 (7.01%)	73/157 (46.50%) vs 139 072/1 085 447 (12.81%)	2/374 (0.53%) vs 723/3 903 189 (0.02%)
RR (95% CI)	**3.72 (1.42–9.83)**	**5.42 (2.06–14.26)**	**9.70 (5.73–16.44)**	**4.54 (2.98–6.93)**	**3.73 (2.87–4.85)**	**3.63 (3.07–4.29)**	**42.76 (12.36–147.92)**
*I* ^2^	Not applicable	Not applicable	Not applicable	Not applicable	Not applicable	Not applicable	90%

Abbreviations: CI, confidence interval; NICU, neonatal intensive care unit; NR, not reported; RR, relative risk.

^a^ Data are presented as number in the group of women >50 years versus number in the group of women <40 years.

^b^ Boldface data are statistically significant.

Findings from indirect meta‐analyses according to maternal age for the risk of stillbirth, cesarean delivery, and maternal mortality are shown in Table 5. The risk of stillbirth and cesarean delivery was significantly higher in women aged >45 years compared with those aged 40–45 years, and in those aged >50 years compared with those aged 45–50 years. The risk of maternal mortality was significantly higher in women aged >50 years compared with those aged 40–45 years (RR 60.40, 95% CI 13.28–274.74).

**TABLE 5:** Indirect meta‐analysis for the risk of stillbirth, cesarean delivery, maternal mortality according to maternal age^a^

	Women >45 years	Women 40–45 years	Relative risk (95% confidence interval)
Stillbirth	105/19 688 (0.53%)	1508/456 596 (0.33%)	**1.61 (1.33–1.97)**
Cesarean delivery	2853/9092 (31.38%)	53 660/186 741 (28.73%)	**1.09 (1.06–1.13)**
Maternal mortality	2/5005 (0.04%)	10/112 952 (0.01%)	4.51 (0.99–20.59)

	Women > 50 years	Women 40–45 years	Relative risk (95% confidence interval)
Stillbirth	4/217 (1.84%)	1508/456 596 (0.33%)	**5.58 (2.11–14.76)**
Cesarean delivery	73/157 (46.50%)	53 660/186 741 (28.73%)	**1.62 (1.37–1.91)**
Maternal mortality	2/374 (0.53%)	10/112 952 (0.01%)	**60.40 (13.28–274.74)**

	Women > 50 years	Women 45–50 years	Relative risk (95% confidence interval)
Stillbirth	4/217 (1.84%)	82/16 739 (0.49%)	**3.76 (1.39–10.17)**
Cesarean delivery	73/157 (46.50%)	1203/4631 (25.98%)	**1.79 (1.50–2.13)**
Maternal mortality	2/374 (0.53%)	31/4631 (0.67%)	0.80 (0.19–3.32)

^a^ Boldface data are statistically significant.

## 4 DISCUSSION

### 4.1 Principal findings

This meta‐analysis aimed to evaluate the risk of maternal and perinatal outcomes in women with advanced maternal age. The primary analyses showed that women aged >40 years had significantly higher risk of stillbirth, perinatal mortality, intrauterine growth restriction, neonatal death, admission to NICU, pre‐eclampsia, preterm birth, cesarean delivery, and maternal mortality compared with those younger than 40 years. These findings were confirmed in subgroup analyses of women aged >45 years and >50 years with even higher RRs (Table 3 and Table 4). Indirect meta‐analyses also showed that the risk of stillbirth, cesarean delivery, and maternal mortality increased with advancing maternal age. The risk ratios for maternal mortality were 3.18, 11.60, and 42.76 in women older than 40, older than 45, and older than 50 years, respectively.

The most important limitation of the meta‐analysis was the inclusion of retrospective non‐randomized studies. The study design of the included studies limited our findings. Different confounders could impact the results of our meta‐analysis. In the group of women with advanced maternal age, most could have had ART‐conceived pregnancies. ART is an independent risk factor for adverse pregnancy outcomes,^44^ and so the risk associated with maternal age per se may have been overestimated. Unfortunately, only a few studies reported separated data for women who underwent ART and therefore these planned subgroup analyses were not feasible.

### 4.2 Implications

Advanced age is a risk factor for female infertility, pregnancy loss, fetal abnormalities, stillbirth, and obstetric adverse outcomes. Infertility increases from 10% at 34 years old to over 85% by the age of 44.^45^ The outcome of in vitro fertilization in women aged 45 years and older who use autologous oocytes is very poor, with an overall delivery rate of 3%.^46^

In recent years the mean age of mothers at first birth has increased, with women delaying childbearing to pursue educational and career goals.^47^, ^48^ As a result, reproductive medicine specialists are facing more patients with age‐related infertility, and maternal‐fetal medicine specialists are treating women with pregnancies complicated by both age and chronic diseases, such as hypertension or diabetes. The media portrayal of a youthful but older woman, able to schedule her reproductive needs and balance family and job, has fueled the myth that “you can have it all,” rarely characterizing the perils inherent in advanced‐age reproduction.^48^ Obstetricians should promote more realistic views of reproductive success according to patient age. The risk, in fact, is that losing time may lead to pregnancy in women over 45 or 50 years of age, using oocyte donation, with unjustifiable risks of maternal and perinatal complications.

### 4.3 Conclusions

In summary, women with advanced maternal age have an increased risk of maternal and perinatal complications. Our meta‐analysis showed that the higher the maternal age the higher the risk of adverse pregnancy outcomes. These data should be used when women with advanced maternal age are counseled regarding their risk in pregnancy.

## CONFLICTS OF INTEREST

The authors report no conflicts of interest.

## AUTHOR CONTRIBUTIONS

GS designed the review, interpreted data, provided the statistical analysis, and reviewed the final version; EG and IS collected data and drafted the manuscript; BI designed the study and drafted the manuscript; VM interpreted data and revised the final manuscript; RV collected data and revised the final manuscript; VB designed the review and drafted and revised the manuscript; and FZ designed the review and revised the final manuscript.

## Data Availability

Data sharing is not applicable to this article as no new data were created or analyzed in this study.
